# Local tissue manipulation *via* a force- and pressure-controlled AFM micropipette for analysis of cellular processes

**DOI:** 10.1038/s41598-018-24255-9

**Published:** 2018-04-12

**Authors:** Phillip Roder, Carsten Hille

**Affiliations:** Department of Physical Chemistry/Applied Laser Sensing in Complex Biosystems (ALS ComBi), Institute of Chemistry, University of Potsdam, Potsdam, Germany

## Abstract

Local manipulation of complex tissues at the single-cell level is challenging and requires excellent sealing between the specimen and the micromanipulation device. Here, biological applications for a recently developed loading technique for a force- and pressure-controlled fluidic force microscope micropipette are described. This technique allows for the exact positioning and precise spatiotemporal control of liquid delivery. The feasibility of a local loading technique for tissue applications was investigated using two fluorescent dyes, with which local loading behaviour could be optically visualised. Thus, homogeneous intracellular distribution of CellTracker Red and accumulation of SYTO 9 Green within nuclei was realised in single cells of a tissue preparation. Subsequently, physiological micromanipulation experiments were performed. Salivary gland tissue was pre-incubated with the Ca^2+^-sensitive dye OGB-1. An intracellular Ca^2+^ rise was then initiated at the single-cell level by applying dopamine *via* micropipette. When pre-incubating tissue with the nitric oxide (NO)-sensitive dye DAF-FM, NO release and intercellular NO diffusion was observed after local application of the NO donor SNP. Finally, local micromanipulation of a well-defined area along irregularly shaped cell surfaces of complex biosystems was shown for the first time for the fluidic force microscope micropipette. Thus, this technique is a promising tool for the investigation of the spatiotemporal effects of locally applied substances in complex tissues.

## Introduction

**Figure 1:**
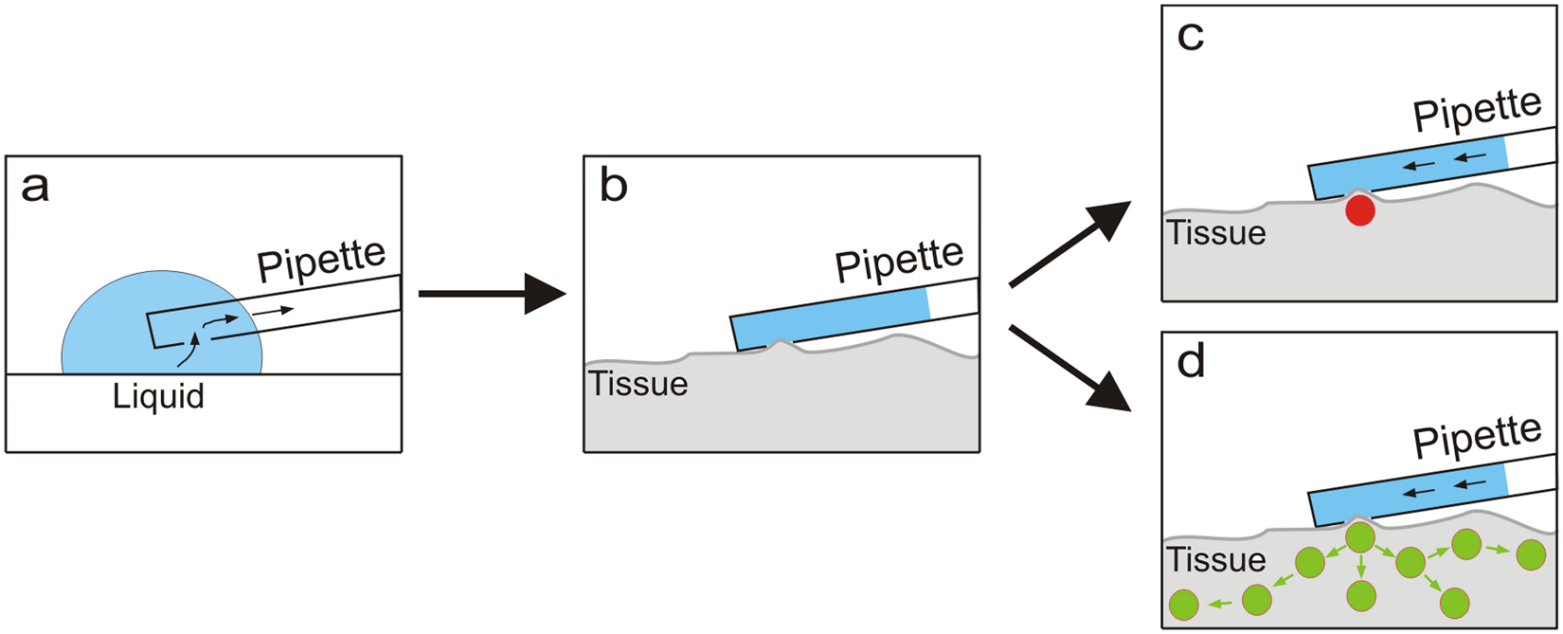
Schematic view of possible applications with the AFM micropipette at living tissue. Schematic illustration of (**a**) the previously published frontloading procedure^18^ and (**b**) the loading position of the micropipette at living tissue with sufficient sealing between micropipette and tissue; estimated observation for (**c**) the CellTracker Red (CTR) and SYTO 9 Green local loading experiments and (**d**) the dopamine and sodium nitroprusside (SNP)-induced local manipulation experiments.

Living cells are the smallest functional unit of all organisms and play an important role due to their complex structure and manifold functions. Individual cells are able to interact with each other resulting in intracellular as well as intercellular signalling in complex tissues. If one of those interactions malfunctions, diseases like metabolic disorders can be observed^1^. Therefore, it is extremely crucial to understand these cell-cell interactions comprehensively. In the past, several different methods were established for manipulating and analysing functions on the cellular level. These include fluorescence microscopy^2,3^, electrophysiology^4^, glass capillaries^5^ and uncaging experiments^6,7^. Since 2009, there is a new method of cellular micromanipulation, which is called ‘fluidic force microscopy’ (FluidFM)^8^. This technique is a combination of atomic force microscopy (AFM) and nanofluidics, which allows for a gentle manipulation and the stimulation of single cells under physiological conditions. There are many different fields of application, such as cell adhesion force measurements^9,10^, single cell injection and extraction^11,12^, and electrochemical layer-by-layer deposition in aqueous environments^13,14^. Currently, most FluidFM experiments are performed only on isolated single cells^15^. However, cell culture-based data cannot reflect the essential physiology of real tissues. In fact, the tissue-specific architecture, mechanical and biochemical cues through an extracellular matrix as well as the cell-cell communication for maintaining the specific functions of the tissue are lost under such simplified conditions^16,17^. Thus, in the present study we have evaluated the FluidFM technique for investigating complex tissues as well. Thereby, a sufficient local sealing of the FluidFM nanofluidics to the region of interest in the tissue is acutely crucial and will be evaluated in the following experiments (Fig. 1). In this context, we apply the previously developed micropipette frontloading technique and the associated cleaning procedure^18^. Especially in physiological experiments, this method is very profitable because some substances, like the biogenic amine dopamine, are not stable for a long time in an aqueous environment. The aim of this study is the examination of inter- and intracellular signalling in living tissue using the FluidFM technique (Fig. 1d). Here, we use the salivary glands of the American cockroach *Periplaneta americana* as an example for complex living tissues, because it is a well-known model system for studying aminergically controlled epithelial ion transport^19–21^. In this way, by combining fluorescence microscopy and fluidic force microscopy, (sub)cellular processes in living tissues can be studied more precisely, making correlative imaging a very powerful research tool^22,23^.

## Results and Discussion

### Local dye loading of single cells in complex tissue

For the local loading experiments, an ideal sealing between the micropipette and the cell membrane of the studied tissue is necessary. Although sufficient sealing could not be observed directly, it could be estimated indirectly by mechanical and optical methods. In the first case, the well-known AFM technology served as a mechanical detection system. *Via* simultaneous deflection and height determination, the position and bending of the cantilever could be monitored online as force-time curves. On the other hand, the sealing could also be observed with fluorescence microscopy by analysing the confinement of fluorescent areas due to the delivered dyes. Therefore, we used the membrane-permeable fluorescent dyes CellTracker Red (CTR) and SYTO 9 Green. After diffusion into cells, the dyes become membrane-impermeable due to esterase activity or nucleic acid binding and will subsequently be trapped intracellularly (Fig. 1c). CTR is an unspecific cell marker, which accumulates throughout the cytosol. CTR exhibits an absorption maximum at λ_ex_ = 577 nm and an emission maximum at λ_em_ = 602 nm^24–26^. In contrast, SYTO 9 can also accumulate in the cytosol, but more effectively, it intercalates into DNA and RNA resulting in a fluorescence enhancement. In comparison to other DNA markers like propidium iodide, SYTO 9 can be applied in living cells. Its absorption maximum is observed at λ_ex_ = 480 nm with an emission maximum at λ_em_ = 500 nm^27,28^.

**Figure 2:**
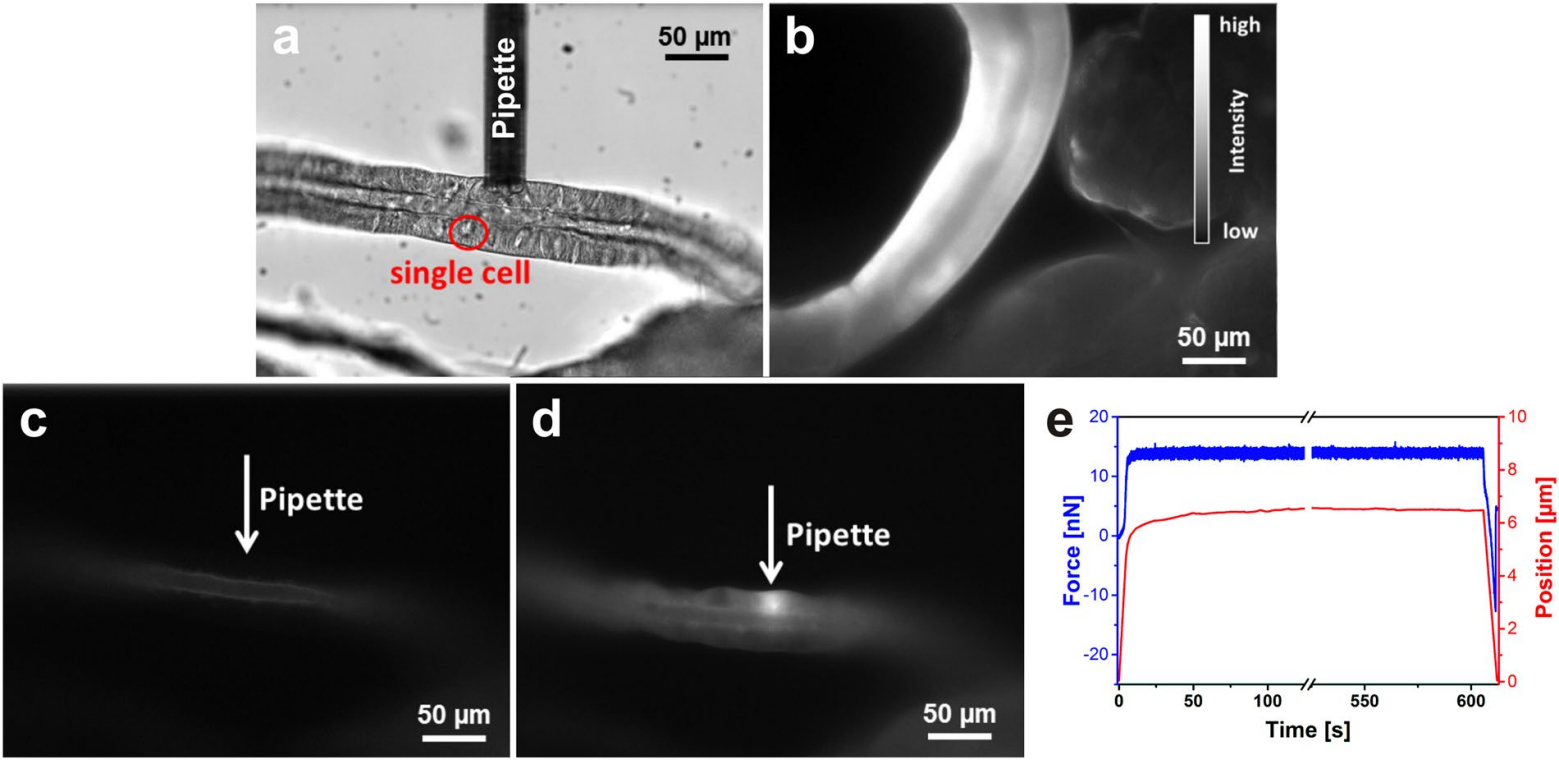
Local dye loading of tissue with CellTracker Red (CTR). (**a**) Bright-field image of salivary duct cells; the red circle marks one single cell. (**b**) Fluorescence image of dye-loaded salivary gland preparation for the bath incubation with 1 µM CTR for 10 min. (**c**) Autofluorescence image of the salivary ducts shown in (**a**) with indication of the consecutive micropipette loading position. (**d**) Fluorescence image of salivary ducts shown in (**a**) after the local dye loading with a micropipette filled with 1 µM CTR. (**e**) Force-time curve during the local loading procedure with the micropipette position; the blue line shows the force and the red line the height position of the micropipette.

In the first set of experiments, the possible dye loading into one single cell of a complex tissue *via* the microchanneled AFM cantilever (micropipette) was evaluated. Therefore, a combination of AFM, frontloading and fluorescence microscopy was used. The selected region, which corresponded to one single cell, was marked in the bright-field image of Fig. 2a. For better comparability of the local dye loading, the living tissue was first loaded from the outside *via* bath incubation for 10 min with 1 µM CTR (Fig. 2b). Because of the bath application, CTR stained all cells along the investigated duct. In addition, only the peripheral cells of the dense acinar tissue were stained as known from the literature. Within the cells, CTR stained the cytosol homogeneously as described in the literature^24^. The corresponding fluorescence image of the unloaded salivary duct from Fig. 2a is shown in Fig. 2c indicating very low intrinsic fluorescence under the chosen experimental conditions. The subsequent loading position of the micropipette was marked (white arrow). At this position, the micropipette was brought in contact with the salivary gland surface with a force of approx. 14 nN for 10 min and the tissue was locally loaded with 1 µM CTR *via* a short pressure pulse of 1 sec. Figure 2d shows the fluorescence image after the local dye loading into one single cell of a duct region, clearly indicating the loading into only one particular cell. An advantage of this staining method compared to the bath incubation is the controlled and highly precise application of dye, requiring a lower number of dye molecules. Here, the delivered micropipette volume was estimated from the calibrated flow rate of 50 fL mbar^−1^ s^−1^ for this micropipette type with a 2 µm opening. Hence, a delivery volume of 50 pL was assumed for a 1 s pressure pulse of 1000 mbar. With this assumption and using a dye concentration of 1 µM, the number of delivery dye molecules resulted in approx. 3 × 10^7^ molecules. In contrast, for the bath incubation a 1 µM dye solution in a bath volume of 1 mL resulted in approx. 6 × 10^14^ molecules for this loading method. Especially when using drugs, this method becomes beneficial in terms of costs as well as tissue survival. Verification of direct contact between the micropipette and the cell surface was also monitored *via* simultaneous force-time curves (Fig. 2e). The blue curve shows the applied force of the cantilever. Thereby, the applied force was constant over time, because force changes were corrected *via* height changes. In addition, within the first few seconds, the approach of the cantilever to the cell membrane could be observed and at the end, a short negative deflection due to an interaction between the micropipette and the tissue during the drive away process could be recognised. The red curve in Fig. 2e shows the corresponding height changes during the measurement. Only a slight height change could be observed, most probably resulting from slight movements of the tissue. However, the stability in the deflection and position was a good sign of a sufficient sealing between the micropipette and the cell membrane during the complete experiment. Otherwise, fluctuating height values would have led to more or less larger gaps between the micropipette and the cell surface.

**Figure 3:**
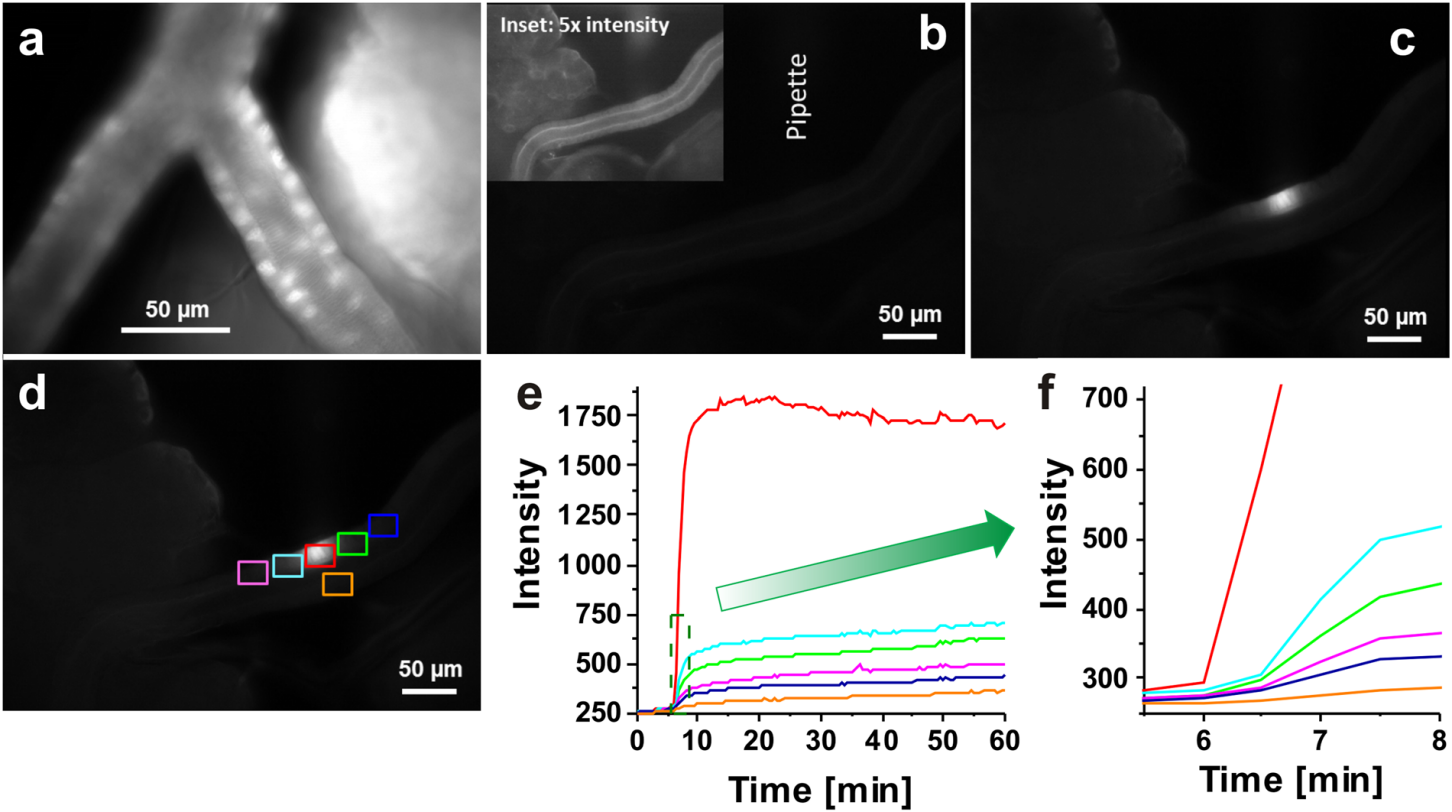
Local tissue loading with SYTO 9 Green. (**a**) Fluorescence image of dye-loaded salivary glands *via* bath incubation with 100 µM SYTO 9 Green for 1 min. (**b**) Autofluorescence image of the examined salivary ducts with indicated micropipette position; inset: 5-fold boosted fluorescence intensity of (**b**) for better visibility. (**c**) Fluorescence intensity image as shown in (**b**), but after the local dye loading procedure. (**d**) Fluorescence intensity image as shown in (**c**), but with six marked regions of interest. (**e**) Time-dependent fluorescence intensity changes within the six analysed regions marked in (**d**). (**f**) Zoom-in of the dashed green box indicated in (**e**).

To investigate the influence of a specifically accumulating dye, the intercalating fluorescence dye SYTO 9 Green was applied in the next step. In this experiment, the usual bath incubation was also compared with the local loading method. Figure 3a shows the fluorescence image of salivary glands, which were bath-incubated in 100 µM SYTO 9 solution for only 1 min. In the ducts, a significant dye accumulation within the cell nuclei could be observed in addition to a low fluorescence background throughout the cytosol. The reasons for this behaviour is the possibility of the dye to intercalate into nucleic acids (RNA and DNA), but with higher binding affinity to DNA leading to higher fluorescence intensities in the nuclei^28^. The observed bright fluorescence signal within the complex acinar tissue could be the result of unspecific dye accumulation in the extracellular matrix of the acini. In contrast, the autofluorescence image of the studied gland preparation together with the micropipette position showed remarkably low background intensity (Fig. 3b). In the inset, the 5-fold enhancement of the autofluorescence image is mapped for better visibility. In the next step, a small amount of 100 µM SYTO 9 solution was sucked into the micropipette and subsequently delivered *via* a short pressure pulse of 2 sec. Thereby, a small area corresponding to one single cell was locally loaded with the dye (Fig. 3c). To illustrate the effect of local loading even better, six regions of interest around the loading position were selected (Fig. 3d) and the fluorescence intensity time traces were plotted (Fig. 3e). In this case, the overall recording time was 60 min, whereas the time period of the micropipette contact comprised only the first 10 min. At around the sixth minute, the fluorescence intensities started to rise in the regions of interest, but to a different extent. To visualise the temporal shift of intensity changes in the six regions, we zoomed in on the green dashed area in Fig. 3e. By doing so, the temporal shift of the individual regions could be clearly visualised and it could be shown that the dye loading initially occurs to the cell, which was directly in contact with the micropipette opening (Fig. 3f). The subsequent slower intensity increase in the neighbouring regions/cells could be the result of insufficient micropipette contact or intercellular dye diffusion. Nevertheless, this local loading method is definitely more targeted than bulk staining and gives methodological access to single cell studies even in complex tissues rather than in isolated cell cultures. Due to the general principle of AFM, only cells at the tissue surface can be manipulated. However, the induced physiological responses can be monitored even in deeper cell layers by inverted fluorescence microscopy. This mainly depends on the optical density or autofluorescence of the tissue type. However, less-scattered near infrared excitation light as used in two-photon microscopy permits deep tissue imaging and penetration depths up to 1 mm^29^.

### Single cell stimulation and induced Ca^2+^ rise in the tissue

After proofing the local loading method, physiological micromanipulation experiments on living tissue preparations were carried out. Thereby, the biogenic amine dopamine was used because its physiological effect is well known for insect salivary glands^30–32^. In insect tissues, the neurotransmitter dopamine can induce an intracellular Ca^2+^ increase by binding to the basolateral G-protein-coupled receptor. Here, the dopamine-activated receptor stimulates the phospholipase C activity leading to hydrolysis of membrane-bound phosphatidylinositol-4,5-bisphosphate into inositol-1,4,5-trisphosphate (IP_3_) and diacylglycerol. Freely diffusing IP_3_ can then bind to IP_3_ receptors at the endoplasmic reticulum leading to Ca^2+^ release into the cytoplasm. Due to gap junctions, IP_3_ can also diffuse into neighbouring cells leading to their activation which can finally be visualised as intercellular Ca^2+^ waves^33–39^. The above-described scenario could also be true for the cockroach salivary acinar cells. However, in the ducts the dopamine-induced intracellular Ca^2+^ rise is a result of the strong Na^+^ reabsorption process from the luminal saliva leading to basolateral Na^+^/Ca^2+^ exchanger activity in the Ca^2+^ entry mode^31^. The function of the resulting Ca^2+^ elevation in the duct cells is still unknown.

**Figure 4:**
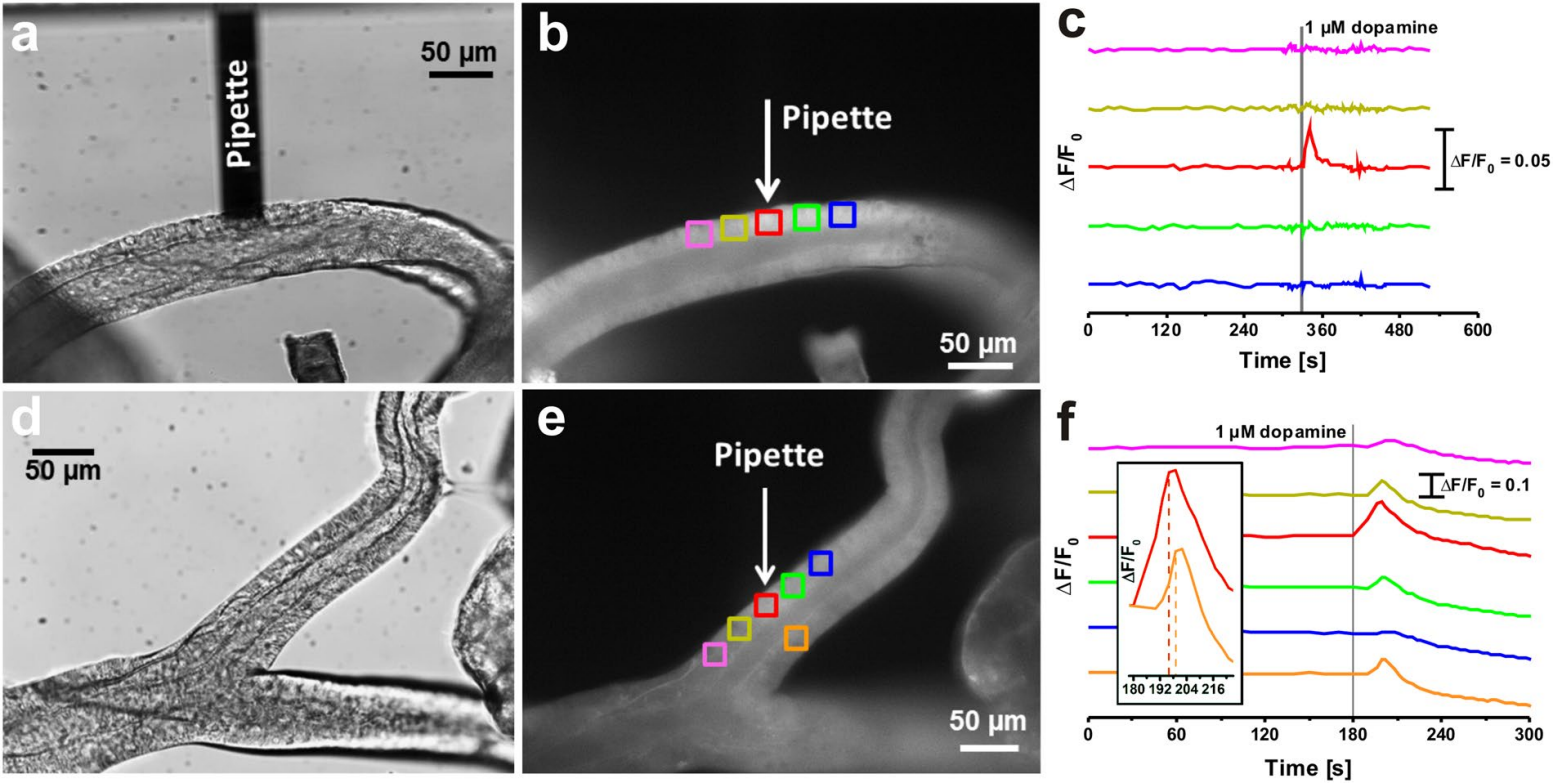
Local dopamine manipulation of OGB-1 preloaded cockroach salivary gland tissue. (**a**) Bright-field image of salivary ducts together with the micropipette used. (**b**) Fluorescence image of an OGB-1 preloaded salivary duct before micromanipulation. (**c**) Time-dependent OGB-1 fluorescence intensity changes within the five regions of interest indicated in (**b**) with a single increase in the red region. F = fluorescence intensity, F_0_ = fluorescence intensity at the beginning. (**d**) Bright-field image of another salivary duct preparation. (**e**) Fluorescence image of the OGB-1 preloaded salivary gland preparation, together with the indicated micropipette position and analysed regions of interest. (**f**) Time-dependent OGB-1 fluorescence intensity changes within the regions of interest indicated in (**e**). The inset indicates an enlarged view of the red and orange regions.

To understand the local effect of dopamine at the duct cells and the subsequent signalling processes in more detail, we started local delivery experiments with dopamine next to the duct cell membrane. Therefore, the salivary glands were preloaded with the Ca^2+^-sensitive dye OGB-1. OGB-1 is a well-established fluorescent dye for Ca^2+^ imaging in the *nM*-range. The dye has an absorption maximum at λ_ex_ = 494 nm and an emission maximum at λ_em_ = 522 nm^40,41^. For physiological experiments, the micropipette was loaded with the physiologically most effective concentration of 1 µM dopamine^32^, brought into contact with the cell membrane and then a short pressure pulse of 1125 mbar s was applied. In this case, we expected to observe a local effect next to the pipette opening directly after the dopamine delivery. In Fig. 4, two examples of local disposal reactions with dopamine are shown. The image acquisition rate was adjusted to 20 min^−1^. In Fig. 4a–c, we could only observe a transient intracellular Ca^2+^ rise in the duct cell next to the pipette opening (red line corresponding to the red region of interest). However, this weak Ca^2+^ rise did not lead to Ca^2+^ changes in the neighbouring cells. In contrast, Fig. 4d–f shows an example of a more complex dopamine response. At first, a transient Ca^2+^ rise could be observed in the duct cell directly in contact with the micropipette (red region in Fig. 4e). Then, time-delayed weak Ca^2+^ signals could be also recognised in the neighbouring regions. With increasing distance of the actual region from the micropipette-stimulated cell, a weaker Ca^2+^ signal was observed. This observation could be a sign of cell-cell communication. In contrast to an intercellular transport, it could also be assumed that this effect was induced due to extracellular dopamine diffusion from the micropipette opening along the duct tissue, assuming insufficient sealing. The dopamine diffusion time *t*_Δx_ could be estimated from the Einstein equation^42,43^ and the known diffusion coefficient of dopamine in buffer near the cell surface (*D* = 8 × 10^−7^ cm^2^ s^−1^)^44,45^ according to

1 $${t}_{{\rm{\Delta }}x}=\frac{{\rm{\Delta }}{x}^{2}}{2D}\,{\rm{.}}$$

The diffusion time of dopamine along a path length of Δ*x* = 50 µm was estimated to *t*_Δx_ ~16 s and did not fit with the detected Ca^2+^ signal from the orange region, which could be seen after 4 s, relative to the first response in the red region. This faster increase in the duct cell within the orange region most probably resulted from intercellular ion diffusion, since the transcellular Na^+^ and Ca^2+^ transport is thought to be part of saliva modification in salivary ducts. According to the diffusion coefficients of Ca^2+^ and Na^+^ (*D* = 5.3 × 10^−6^ cm^2^ s^−1^ for Ca^2+^ ^46^ and *D* = 1.2 × 10^−5^ cm^2^ s^−1^ for Na^+^ ^47^), their intracellular diffusion time of Δ*x* = 50 µm was estimated to *t*_Δx_ ~1–3 s, fitting well to the observed data in Fig. 4f.

### NO release in gland tissue after local micromanipulation

Nitric oxide (NO) is also an important second messenger in living cells and is involved in many physiological processes like neurotransmission, immune defence and regulation of cardiovascular homeostasis. For this reason, it also plays an essential role in the study of heart diseases^48–50^. NO is a short-lived free radical^51^ and can be synthesised from *L*-arginine *via* the enzyme nitric oxide synthase (NOS), of which three isoforms are known. The neuronal and endothelial forms of NOS are dependent on Ca^2+^, whereas the inducible NOS is not. One of the suggested actions of NO is its activation of soluble guanylyl cyclases leading to a cGMP rise, influencing protein kinase activities downstream^52^. Stimulus-induced NO production in salivary gland cells has been suggested from pharmacological studies, but its functions are still under investigation^53^. One reason is the lack of adequate fluorescent dyes for dynamic NO recordings. Here, we used the fluorescent dye 4-amino-5-methylamino-2′,7′-diaminodifluorofluorescein (DAF-FM) diacetate. In this context, NO release could be detected in complex cockroach salivary gland tissue for the first time. In the presence of a NO radical, the dye reacts with NO^+^ by nitrosation and dehydration to form the stable and highly fluorescent triazolofluorescein. NO^+^ is formed from N_2_O_3_ through former autoxidation of NO. Finally, NO is irreversibly bound to the DAF-FM molecule and no further changes in NO concentrations can be monitored. DAF-FM absorbs at λ_ex_ = 495 nm and emits at λ_em_ = 515 nm^54,55^. As a NO donor, we used sodium nitroprusside (SNP) to study the diffusion behaviour of locally released NO in a complex tissue. For physiological experiments, SNP concentrations in the *µM* to *mM*-range are used. Furthermore, the NO donor effectivity is increased by light^56–59^.

For the local NO experiments, we loaded the micropipette with 20 mM SNP. The micropipette was positioned directly at the cell surface of the acinar tissue and a short pressure pulse of 1125 mbar·s combined with a 5 s illumination period of white light was given. For comparison, a bulk bath application of 5 mM SNP to the salivary acinar tissue occurred approx. 3 min after the local delivery experiment, which was additionally activated after 7 min by a short light pulse of 5 s.

**Figure 5:**
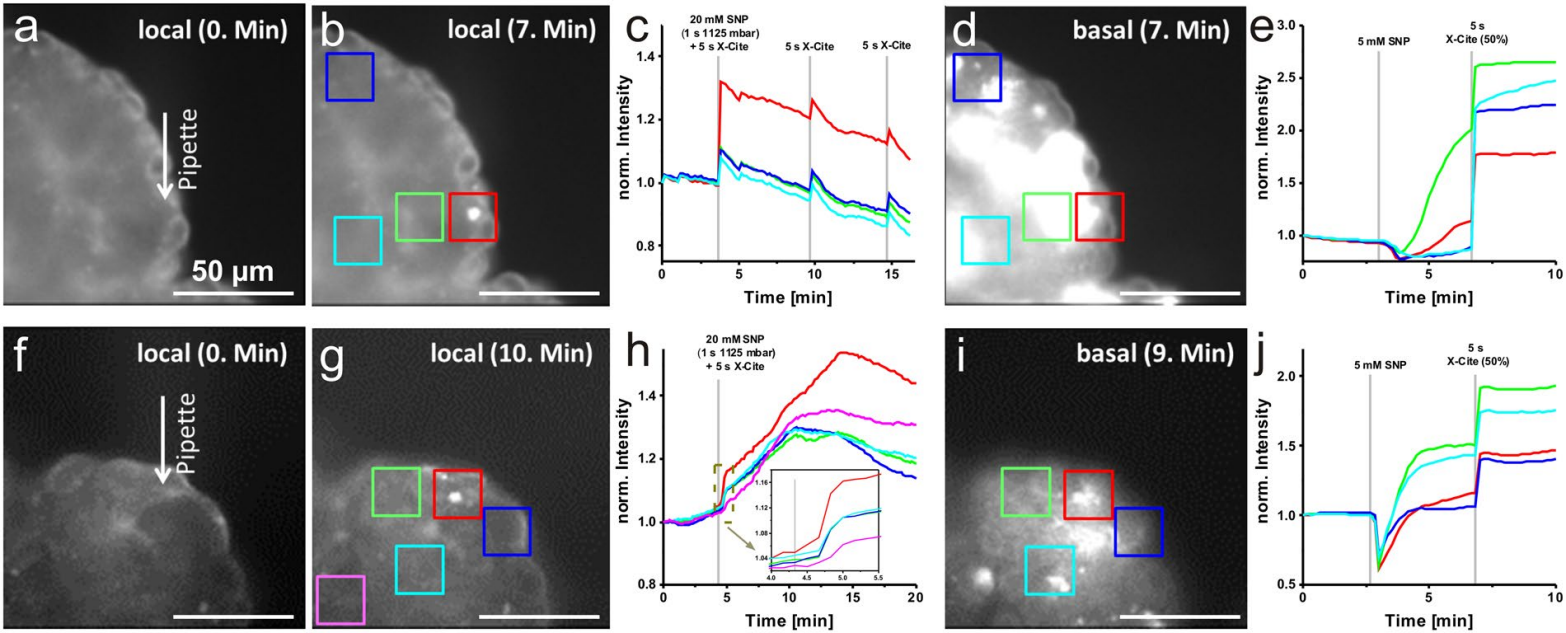
Local SNP manipulation of DAF-FM diacete preloaded cockroach salivary gland tissue. Fluorescence images of salivary acinar tissue (**a**) before and (**b**) after the local delivery of 20 mM SNP. The position of the pipette opening is marked with an arrow. (**c**) Time-dependent DAF-FM fluorescence intensity trends within the four marked regions in (**b**). (**d**) Fluorescence image of the examined acinar tissue of (**a**) after the bulk bath application of 5 mM SNP and its activation with a 5 s white light pulse. (**e**) Fluorescence intensity time traces of the four marked regions in (**d**). Fluorescence images of another salivary acinar tissue preparation (**f**) before and (**g**) after the local manipulation with 20 mM SNP. (**h**) Time-dependent DAF-FM fluorescence intensity trends within the five marked areas in (**g**). The dashed rectangle is enhanced in the zoom-in graph, unravelling a slight temporal shift. (**i**) Corresponding fluorescence image of the acinar tissue preparation in (**f**) after the bulk bath application of 5 mM SNP and 5 s light pulse. (**j**) Fluorescence intensity time traces of the four marked regions in (**i**). Scale bars = 50 µm.

Two representative local NO experiments together with the corresponding control experiments are shown in Fig. 5. The fluorescence images of DAF-FM-loaded salivary acinar tissue together with the micropipette position before and after the local SNP delivery are shown in Fig. 5a and b, indicating a significant increase in the fluorescence intensity next to the micropipette opening, which in turn indicates a sufficient NO production from the delivered NO donor SNP. Since DAF-FM was accumulated intracellularly, the produced NO had to diffuse through the acinar cell membrane. The local NO effect could be analysed for different regions of interest (Fig. 5c). Besides the large intensity increase directly in the cell that was in contact with the micropipette, small intensity rises could also be observed in the other regions of the investigated acinar tissue. This effect is more a light-induced, unspecific reaction of DAF-FM or the cellular autofluorescence than an effect of NO diffusion through neighbouring cells. An indication for this assumption could be obtained from the identical time-dependent changes in the intensities after the local delivery (see Fig. 5c). The same observation was visible after application of a second and third light pulse (Fig. 5c). Figures 5d and e show the effects of SNP bath application, indicating a stronger and large-area effect compared to the local SNP delivery. Especially after application of a light pulse, a huge intensity rise could be observed. Therefore, for sufficient NO production from the NO donor SNP, a light pulse is beneficial. However, this experiment proved the sufficient accumulation of NO-sensitive dye in almost all cells of the investigated acinar tissue preparation.

In Fig. 5f–h, another example of successful local SNP delivery is shown. Similar to the first example, a local DAF-FM fluorescence intensity increase in cells next to the micropipette opening could be observed directly after the SNP release (Fig. 5g). Here, in addition to a rapid DAF-FM intensity increase within the first minute after the stimulus (see zoom-in in Fig. 5h), a long-time increase in the intensity over a few minutes could be observed (Fig. 5h). When analysing different regions of interest, a temporal shift in the intensity changes could also be recognised. In addition to an incomplete sealing of the micropipette, the small temporal shift of the signal could also be evidence of a cell-cell interaction or rather intercellular NO diffusion. Figures 5i,j show the positive control effect of an SNP bath application, leading to similar trends as shown in Fig. 5e.

## Conclusions

Nowadays, physiological studies at the single cell level are of great interest, in order to understand cellular processes in the context of complex tissues/organs in more detail. The recently developed fluidic force microscopy seems to be a very powerful tool for performing such studies. This technique allows for the exact positioning and precise spatiotemporal control of the delivery of physiologically relevant substances. Here, we have represented applications for the frontloading method of the pressure-controlled microchanneled AFM micropipette at complex tissue surfaces. We were able to show highly localised fluorescent dye delivery, as well as locally induced physiological effects on living complex tissue. However, it also turned out that technical parameters such as the applied pressure or delivery time do have to be precisely adjusted for each biosystem and/or applied substance.

Starting from the results presented here, transepithelial processes in the salivary glands can now be investigated in more detail. For instance, this method could be used to study the stimulus-induced Na^+^ transport after the local micropipette-driven application of the ionophore ionomycin. Ionomycin provokes an intracellular Ca^2+^ increase leading to a time-shifted Na^+^ rise in the salivary gland cells^60^. This application would be a single-cell-level continuation of a previous study on the Na^+^ transport in living cockroach salivary glands using the Na^+^-sensitive fluorescent dye Asante NaTRIUM Green-2 (ANG-2)^61^. Alternatively, this method could also be used to study the diffusion behaviour of fluorescent markers, now having control over the investigated region of interest as well as the number of applied marker molecules. In combination with single molecule detection methods such as fluorescence correlation spectroscopy, the diffusion behaviour can be specifically investigated at a high spatial resolution. Such studies could significantly contribute to the analysis of subcellular organisation in living cells^62^.

## Methods

### Reagents and solutions

Different fluorescent dyes were used to examine the living tissue. For the local sealing experiments, the fluorescent dyes CellTracker Red (CTR) and SYTO 9 Green (both from Life Technologies, Darmstadt, Germany) were applied. CTR was dissolved in dimethyl sulfoxide (DMSO) to a stock solution of 1 mM, and the final concentration in physiological saline was 1 µM. SYTO 9 was delivered in a 5 mM DMSO stock solution and was diluted with buffer to final concentrations between 10–100 µM. Oregon Green 488 BAPTA-1 (OGB-1) (Life Technologies, Darmstadt, Germany) was used as a Ca^2+^-sensitive fluorescent dye for physiological Ca^2+^ experiments. The 2 mM stock solution in DMSO was diluted with physiological saline into the final concentration of 6.67 µM. For the other physiological experiments, the NO-sensitive fluorescent dye 4-Amino-5-Methylamino-2′,7′-Difluorofluorescein (DAF-FM) diacetate (Life Technologies, Darmstadt, Germany) was used in a final concentration of 10 µM, prepared from a 3 mM stock solution in DMSO^54,55^.

Physiological saline was composed of 160 mM NaCl, 10 mM KCl, 2 mM CaCl_2_, 2 mM MgCl_2_, 10 mM glucose and 10 mM Tris at pH 7.4^29^. The pH value was adjusted with HCl. A 10 mM dopamine (Sigma-Aldrich, Deisenhofen, Germany) stock solution in double distilled water was freshly prepared before the physiological experiments. The final concentration in physiological saline was 1 μM. A 1 M stock solution of the NO-releasing substance sodium nitroprusside (SNP) (Riedel-de Haën, Seelze, Germany) was diluted in physiological saline to a final concentration of 20 mM directly before an experiment.

For the micropipette cleaning, 12% sodium hypochlorite (NaOCl) stock solution (Carl Roth, Karlsruhe, Germany) was diluted with double distilled water to a 4% NaOCl solution.

### Combined FluidFM probe and fluorescence microscope setup

For our setup, we used a FlexAFM scan head (Nanosurf, Langen, Germany), which was connected with a microchanneled AFM micropipette (Cytosurge, Zurich, Switzerland) *via* a probe holder. The scan head and the nanofluidics were triggered by the C3000 controller (Nanosurf, Langen, Germany) and the ARYA software ver. 1.0.45 (Cytosurge, Zurich, Switzerland). The AFM micropipette was composed of silicon nitride, had a reflective gold-chrome coating on the back side, and possessed a length of 200 µm, a width of 36 µm and a tip opening of 2 µm. *Via* a silicone tube and a connector, the micropipette was combined with a pressure controller. In combination with an external pressure controller software ver. 1.2.5 (Cytosurge, Zurich, Switzerland), the pressure was set between −800 mbar (low pressure) and 1125 mbar (high pressure). The monitoring of the cantilever height position was controlled by a standard near-infrared AFM laser and a photodiode detection system^8^. The detected electronic signal (mV) was converted into the force (nN) using the spring constant (N/m) and the measured sensitivity (m/V). For concurrent fluorescence microscopy, a Zeiss Axio Observer Z1 inverted microscope (Carl Zeiss, Jena, Germany) was modified with a specifically adapted AFM sample stage (Nanosurf, Langen, Germany) in order to combine the AFM scan head with the fluorescence microscope stage. The microscope was equipped with the objectives Zeiss Fluar 20×/NA 0.75 and Zeiss Fluar 40×/NA 0.75. In order to separate the excitation and emission light, two different filter cubes working at λ_ex_ = 485 nm ± 15 nm and λ_em_ = 535 nm ± 25 nm, or at λ_ex_ = 565 nm ± 15 nm and λ_em_ = 630 nm ± 37.5 nm were used. Image acquisition was carried out *via* a CoolSnap HQ^2^ CCD camera or a Cascade 128^+^ EMCCD camera for the NO-experiments (both from Photometrics, Arizona, USA), and the software MetaMorph ver. 7.1 (Molecular Devices, Sunnyvale, USA) after excitation with an X-Cite short-arc lamp (Visitron Systems, Puchheim, Germany). Data and image analysis as well as the graphical presentation were performed using Origin 9.1 G (Origin Lab Corp., Northampton, USA), ImageJ ver. 1.51e and CorelDRAW X3 (Corel, Munich, Germany).

### Frontloading and cleaning procedures

The micropipette was loaded with substances *via* the previously developed frontloading procedure^18^. In our present experiments, we used volumes of about 25 µL containing the target substance. The micropipette was filled with the required solution *via* a low pressure of −800 mbar for a few seconds. After the loading, the micropipette was cleaned from the outside with 4% NaOCl solution or water, depending on the target substance used. After an experiment, the residual solution in the micropipette was removed with a high pressure of 1125 mbar. Subsequently, the micropipette was submerged into a drop of the cleaning solution (e.g. NaOCl or water) and the inside of the micropipette was rinsed through repeated loading (low pressure −800 mbar) and release (high pressure 1125 mbar) cycles. All target and cleaning solutions were filtered (0.1 µm syringe filter) to prevent plugging of the microchannel due to small amounts of residual solid matter.

### Tissue preparation

A colony of the American cockroach P. *americana* was reared at the Department of Animal Physiology (University of Potsdam) at 27 °C under a light/dark cycle of 12 h: 12 h with free access to food and water. For the experiments, only male adults were taken. Salivary glands were dissected in physiological saline as described previously^19^. Small lobes of the salivary gland consisting of a branched duct system and several acini were used for the micromanipulation experiments.

For the physiological Ca^2+^ experiments, freshly dissected cockroach salivary glands were first loaded for 60 min with a hypotonic physiological saline (75% physiological saline + 25% double distilled water) containing 6.67 µM of the Ca^2+^-sensitive fluorescent dye OGB-1. For the physiological NO experiments, the prepared tissue was loaded with 10 µM DAF-FM diacetate in hypotonic saline for 60 min.

### Data availability

The datasets generated during and/or analysed during the current study are available from the corresponding author upon reasonable request.
